# Basic Reproduction Number of the 2019 Novel Coronavirus Disease in the Major Endemic Areas of China: A Latent Profile Analysis

**DOI:** 10.3389/fpubh.2021.575315

**Published:** 2021-09-14

**Authors:** Honglv Xu, Yi Zhang, Min Yuan, Liya Ma, Meng Liu, Hong Gan, Wenwen Liu, Gillian Gianna Anne Lum, Fangbiao Tao

**Affiliations:** ^1^School of Medicine, Kunming University, Kunming, China; ^2^Key Laboratory of Population Health Across Life Cycle, Ministry of Education of the People's Republic of China, Anhui Medical University, Hefei, China; ^3^Department of Maternal, Child and Adolescent Health, School of Public Health, Anhui Medical University, Hefei, China; ^4^School of Health Service Management, Center for Big Data Science in Health, Anhui Medical University, Hefei, China; ^5^The Colonial War Memorial Hospital, Suva, Fiji

**Keywords:** 2019 novel coronavirus disease, basic reproduction number, latent categories, trends, epidemiology

## Abstract

**Objective:** The aim of this study is to analyze the latent class of basic reproduction number (*R*_0_) trends of the 2019 novel coronavirus disease (COVID-19) in the major endemic areas of China.

**Methods:** The provinces that reported more than 500 cases of COVID-19 till February 18, 2020 were selected as the major endemic areas. The Verhulst model was used to fit the growth rate of cumulative confirmed cases. The *R*_0_ of COVID-19 was calculated using the parameters of severe acute respiratory syndrome (SARS) and COVID-19. The latent class of *R*_0_ was analyzed using the latent profile analysis (LPA) model.

**Results:** The median *R*_0_ calculated from the SARS and COVID-19 parameters were 1.84–3.18 and 1.74–2.91, respectively. The *R*_0_ calculated from the SARS parameters was greater than that calculated from the COVID-19 parameters (*Z* = −4.782 to −4.623, *p* < 0.01). Both *R*_0_ can be divided into three latent classes. The initial value of *R*_0_ in class 1 (Shandong Province, Sichuan Province, and Chongqing Municipality) was relatively low and decreased slowly. The initial value of *R*_0_ in class 2 (Anhui Province, Hunan Province, Jiangxi Province, Henan Province, Zhejiang Province, Guangdong Province, and Jiangsu Province) was relatively high and decreased rapidly. Moreover, the initial *R*_0_ value of class 3 (Hubei Province) was in the range between that of classes 1 and 2, but the higher *R*_0_ level lasted longer and decreased slowly.

**Conclusion:** The results indicated that the overall *R*_0_ trend is decreased with the strengthening of comprehensive prevention and control measures of China for COVID-19, however, there are regional differences.

## Introduction

Of particular concern is the 2019 novel coronavirus disease (COVID-19) outbreak in Wuhan, Hubei province at the end of 2019, it is quickly spread to all over the country of China (1, 2). COVID-19 is an infectious disease caused by 2019-nCoV that can be transmitted through droplets, aerosols, and contact (3, 4). The main clinical features of the case are fever, dry cough, and fatigue. There are also mild features as well as asymptomatic pathogen carriers (5, 6). Evidence demonstrates that COVID-19 is extremely infectious even during the incubation period, and the entire population is susceptible (7, 8). It has a higher case-fatality rate and poses great threats to public health, and as a result it has attracted enormous concerns (9). According to previous studies, in the USA, the case-fatality rate for COVID-19 varies markedly by age, ranging from 0.3 death per 1,000 cases among patients aged from 5 to 17 years to 304.9 deaths per 1,000 cases among patients aged 85 years or older in the USA (10). Among the hospitalized patients in the intensive care units, the case fatality increases up to 40% (10). In addition, Yang et al. (9) reported that Wuhan had higher case fatality than other cities. Overall, a difference of the case-fatality rates was observed between different age groups, cities, and counties, at the rates more than 15% (11). At present, COVID-19 has become widespread around the globe (12, 13). For a new infectious disease, it is important to identify the epidemic characteristics and transmission dynamics of the disease, to implement appropriately for the prevention and control measures (14).

It is generally known that the basic reproduction number (*R*_0_) is an important parameter that studies the dynamics of infectious disease transmission by describing the ability to spread an infectious source (15, 16). *R*_0_ is defined as the average number of secondary cases produced by an infected subject over his/her infectious period in a susceptible and an uninfected population (17). There has been an increasing concern that the *R*_0_ of COVID-19 is used to assess the spread of infectious diseases, to predict epidemic trends and to evaluate the effectiveness of the prevention and control measures that have been established (18, 19). Although a few previous studies have investigated the *R*_0_ of COVID-19, the results of these previous studies on the *R*_0_ of COVID-19 were inconsistent (20–23). For instance, Wu et al. (20) used susceptible-exposed-infectious-recovered metapopulation model to analyze the confirmed cases from December 31, 2019 to January 28, 2020, the results reported that the *R*_0_ of COVID-19 was 2.68 (95%CI: 2.47–2.86); Zhao et al. (21) used an intrinsic growth rate (*γ*) to the analyzed *R*_0_ of the confirmed cases from January 10, 2020 to January 24, 2020, the results reported that the *R*_0_ of COVID-19 was in the range of 2.24–3.58 (21). Moreover, there is no research to explore the variance in the *R*_0_ of COVID-19 in the different regions of China. Consequently, this study analyzed the latent class of *R*_0_ of COVID-19 in the major endemic areas of China that reported more than 500 cases. The results of this study can evaluate the effectiveness of the prevention and control measures of China against COVID-19 to a certain extent and provide a support for previously related studies and a reference for other countries to learn from the prevention and control measures of China. In addition, it provides a basis for further prevention and control of COVID-19 and other emerging infectious diseases.

## Materials and Methods

### Materials

The major endemic areas of COVID-19 were defined as the areas where the cumulative number of cases of COVID-19 with more than 500 has been diagnosed within 24 h on February 18, 2020. These areas include Guangdong Province, Jiangxi Province, Hunan Province, Chongqing Municipality, Sichuan Province, Hubei Province, Anhui Province, Zhejiang Province, Jiangsu Province, Henan Province, and Shandong Province. We collected the cumulative number of cases of COVID-19 from official health commission websites, with the data on the main prevention and control measures for COVID-19 within these areas and sourced from provincial government websites.

### Methods

The Verhulst model was used to fit the growth rate of the cumulative number of cases (24, 25). The Verhulst curvilinear equation is as follows:


(1) $$y=\frac{a}{1+(\frac{a}{b}-1)exp(-cx)}$$

(2) $$r=\frac{d_{y}}{yd_{x}}$$

where *a, b*, and *c* are the parameter constants and *r* is the growth rate. We calculated the *R*_0_ of COVID-19 using the following equation (26):


(3) $$R_{0}=(1+r\times IP)\times (1+r\times SI)$$

where *r* is the growth rate, IP is the incubation period, SI is the serial interval from the onset of case i to the infection of case ii. We calculated *R*_0_ based on the IP and SI of COVID-19 (reported by the Chinese Center for Disease Control and Prevention) and severe acute respiratory syndrome (SARS). The IP and SI of COVID-19 are 5.2 and 7.5, respectively (4). The IP and SI of SARS are 6.0 and 8.4, respectively (27). We described the changing trend of *R*_0_ with time in each province, and analyzed the latent class according to the trend of *R*_0_. Moreover, we gathered the major prevention and control measures for COVID-19 in each endemic area.

### Statistical Analyses

All statistical analyses were performed using R (R-3.5.1, R Core Team) and Mplus (Mplus version 7.4). The methods used for an analysis included the descriptive statistical analysis method and the model estimation method. The Verhulst model was used to fit the growth rate of the cumulative number of cases and to calculate *R*_0_ in the R software. We used the range between minimum and maximum values, the 25th percentile (*P*_25_), the 50th percentile (*P*_50_), and the 75th percentile (*P*_75_) to describe the distribution of *R*_0_. The Wilcoxon signed-rank test was used to compare the differences of *R*_0_ calculated from the COVID-19 parameter and the SARS parameter. A latent profile analysis (LPA) was used to analyze the latent class of the *R*_0_ trend in the Mplus software.

## Results

### The Trend of the Cumulative Number of Cases

**Figure 1:**
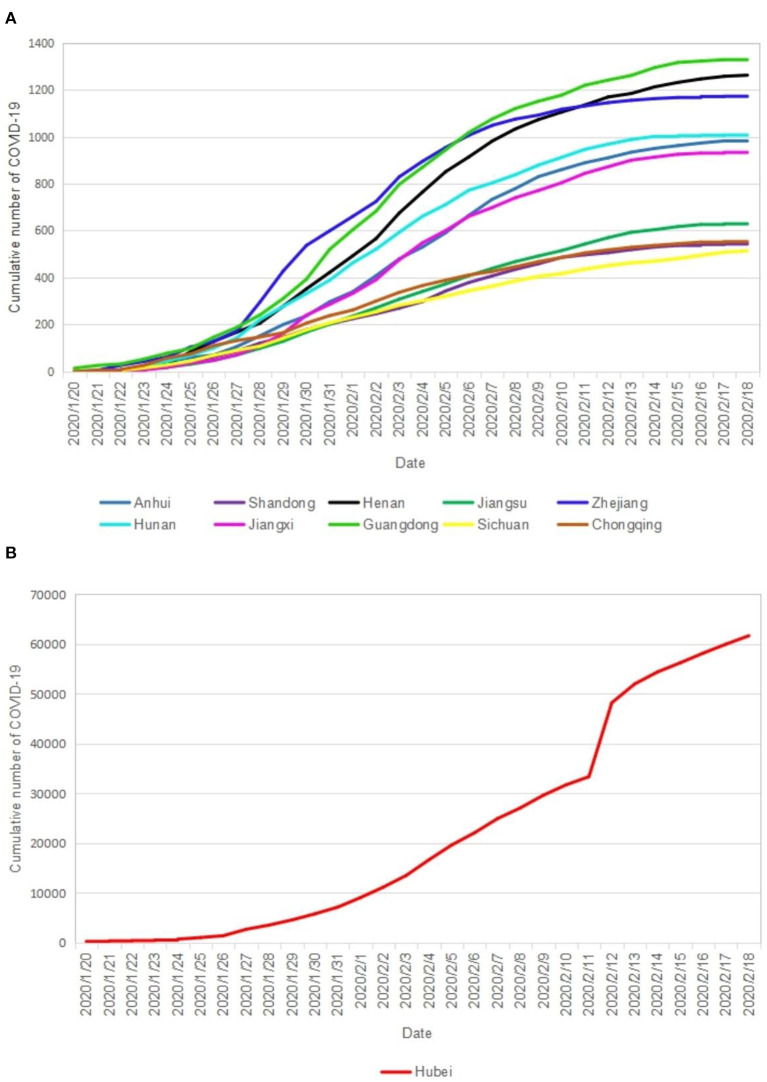
The cumulative number of COVID-19 in the major epidemic areas of China. **(A)** Cumulative number of COVID-19 in each province; **(B)** Cumualtive nember of COVID-19 everyday.

Figure 1 shows the trend of the cumulative number of COVID-19 It shows an S-shaped growth. The Hubei Province has the largest cumulative number of cases and the fastest growth. The trends of the cumulative number of cases in several other provinces except the case of Hubei Province were observed in the phenomenon of classification. The number of cases in class 1 (Henan Province, Zhejiang Province, and Guangdong Province) was relatively large and has increased rapidly. The number of cases in class 2 (Anhui Province, Hunan Province, and Jiangxi Province) and the growth rate were at a medium level. The number of cases in class 3 (Shandong Province, Jiangsu Province, Sichuan Province, and Chongqing Municipality) and the growth rate were relatively low. In general, the major endemic areas except the case of Hubei Province of COVID-19 in China reached the highest peaks of the “S” curve around February 15, 2020.

### The Growth Rate Trend of the Cumulative Number of Cases

**Figure 2:**
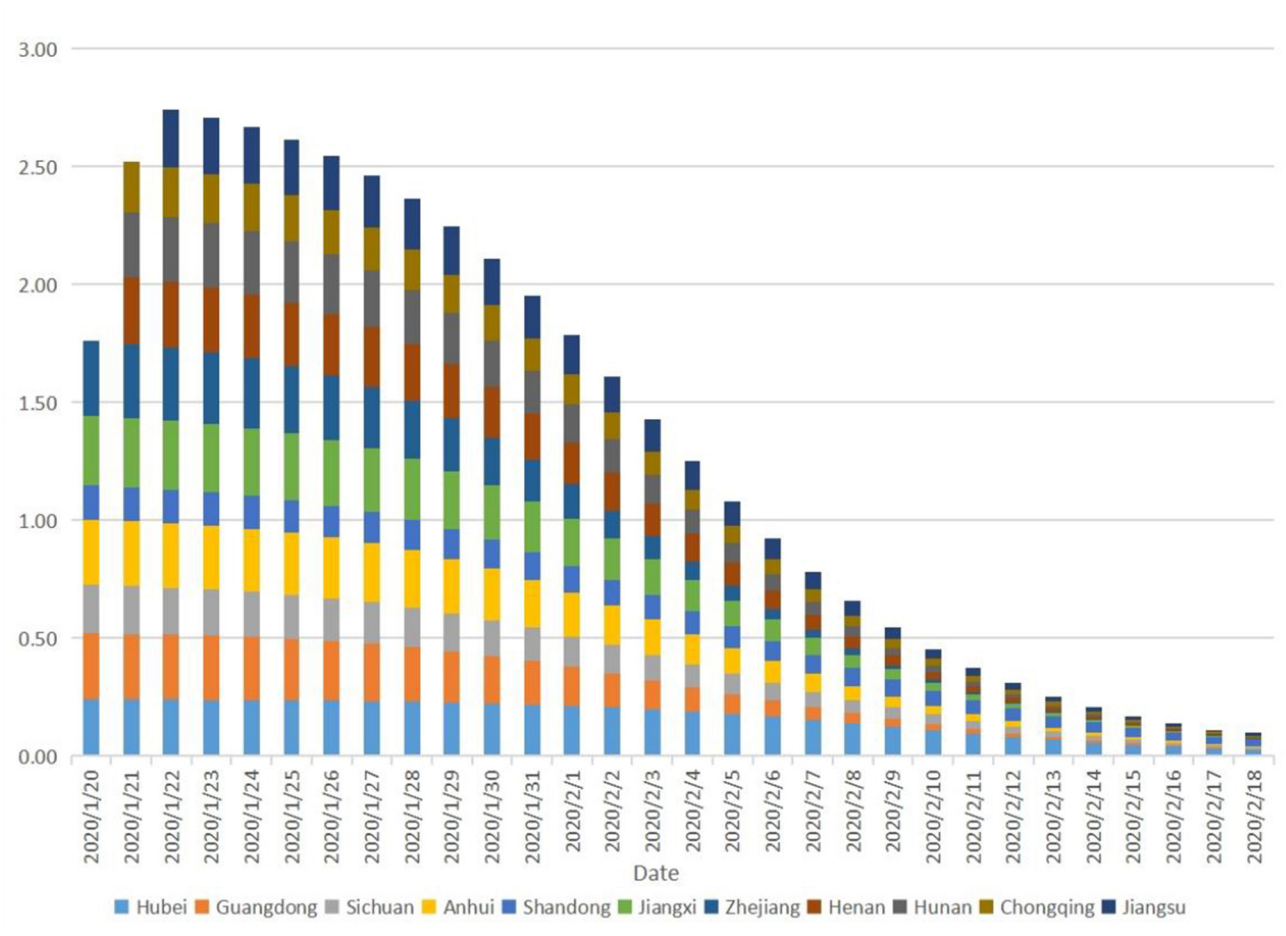
The growth rate of cumulative number of COVID-19.

The growth rate of the cumulative number of COVID-19 in Hubei Province is 0.03–0.20. Especially, the growth rate of 0.24 continued until January 26, 2020, whereas the growth rate of 0.23–0.22 continued until January 31, 2020. Then, the growth rate shows a clear downward trend in Hubei Province. The initial growth rates (0.25–0.32) of all other provinces except the cases of Chongqing Municipality, Sichuan Province, and Shandong Province (the growth rates were 0.14–0.20) were higher than those of Hubei Province, however, these rates started to decline rapidly after 2–3 days (Figure 2).

### The R_0_ Trend of COVID-19

**Figure 3:**
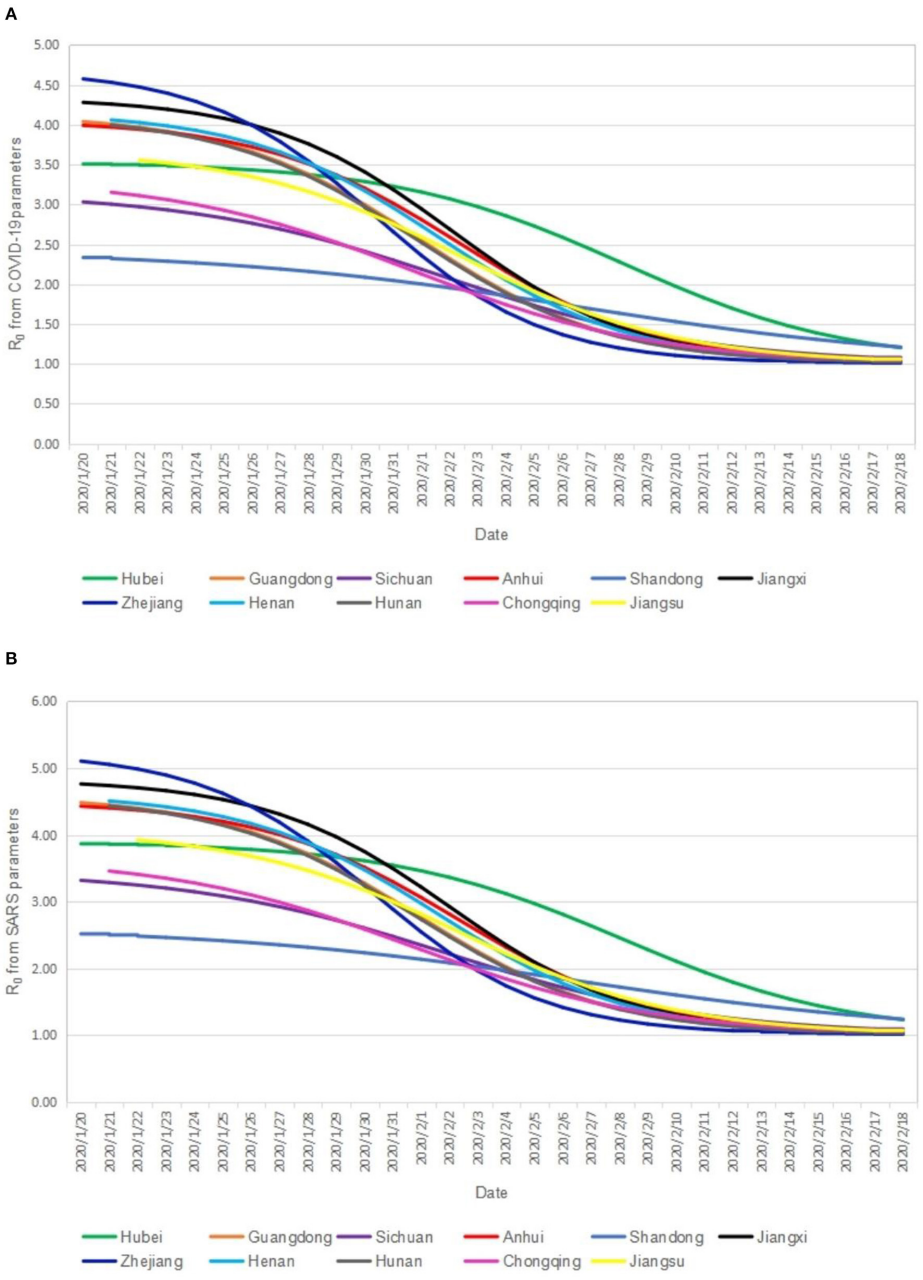
The R_0_ trend from COVID-19 and SARS parameters. **(A)** Cumulative number of COVID-19 in each province; **(B)** Cumualtive nember of COVID-19 everyday.

Table 1 shows the distribution of *R*_0_. The median *R*_0_ calculated from the SARS parameters is 1.84–3.18, and the *R*_0_ calculated from the COVID-19 parameters is 1.74–2.91. Overall, the former is greater than the latter (*Z*=−4.782 to −4.623, *p* < 0.01). Figure 3 shows the *R*_0_ trend collected from the COVID-19 and SARS parameters. The trends of *R*_0_ calculated from the two parameters are basically consistent. It showed a gradual downward trend of *R*_0_ in each province from January 20, 2020 to February 18, 2020. The *R*_0_ in Hubei Province showed a slow decline before January 31, 2020, then showing a significant downward trend. The initial *R*_0_ in all other provinces except the cases of Chongqing Municipality, Sichuan Province, and Shandong Province was higher than that of Hubei Province, however, but the declining rate of *R*_0_ in all other provinces except the cases of Chongqing Municipality, Sichuan Province, and Shandong Province is higher than that of Hubei Province. As of February 18, 2020, except the cases of Hubei Province (*R*_0_ = 1.20) and Shandong Province (*R*_0_ = 1.21), *R*_0_ (1.01–1.06) were approximately equal to 1 in all other provinces.

**Table 1:** The distribution of *R*_0_ of 2019 novel coronavirus disease (COVID-19).

**Province**	***R***_**0**_ **from SARS parameters**				**R**_**0**_ **from COVID-19 parameters**				***Z***	***P***
	**Range**	***P*_**25**_**	***P*_**50**_**	***P*_**75**_**	**Range**	***P*_**25**_**	***P*_**50**_**	***P*_**75**_**		
Hubei	1.23–3.87	1.90	3.18	3.76	1.20–3.51	1.80	2.91	3.41	−4.782	<0.01
Guandong	1.02–4.48	1.16	2.13	3.93	1.02–4.04	1.15	2.00	3.56	−4.782	<0.01
Sichuan	1.07–3.32	1.28	2.01	2.94	1.06–3.03	1.25	1.90	2.71	−4.782	<0.01
Anhui	1.04–4.43	1.24	2.44	4.03	1.03–3.99	1.22	2.26	3.65	−4.782	<0.01
Shandong	1.24–2.51	1.53	1.99	2.36	1.21–2.33	1.47	1.88	2.20	−4.782	<0.01
Jiangxi	1.03–4.76	1.21	2.49	4.34	1.03–4.28	1.19	2.31	3.92	−4.782	<0.01
Zhejiang	1.01–5.10	1.08	1.85	4.25	1.01–4.57	1.07	1.75	3.84	−4.782	<0.01
Henan	1.03–4.51	1.19	2.19	3.96	1.03–4.06	1.17	2.05	3.59	−4.703	<0.01
Hunan	1.02–4.43	1.15	2.00	3.78	1.02–4.00	1.13	1.88	3.43	−4.703	<0.01
Chongqing	1.05–3.46	1.20	1.84	2.93	1.04–3.15	1.18	1.74	2.69	−4.703	<0.01
Jiangsu	1.05–3.92	1.25	2.12	3.43	1.05–3.56	1.22	1.99	3.13	−4.623	<0.01

### The Latent Class of R_0_

**Figure 4:**
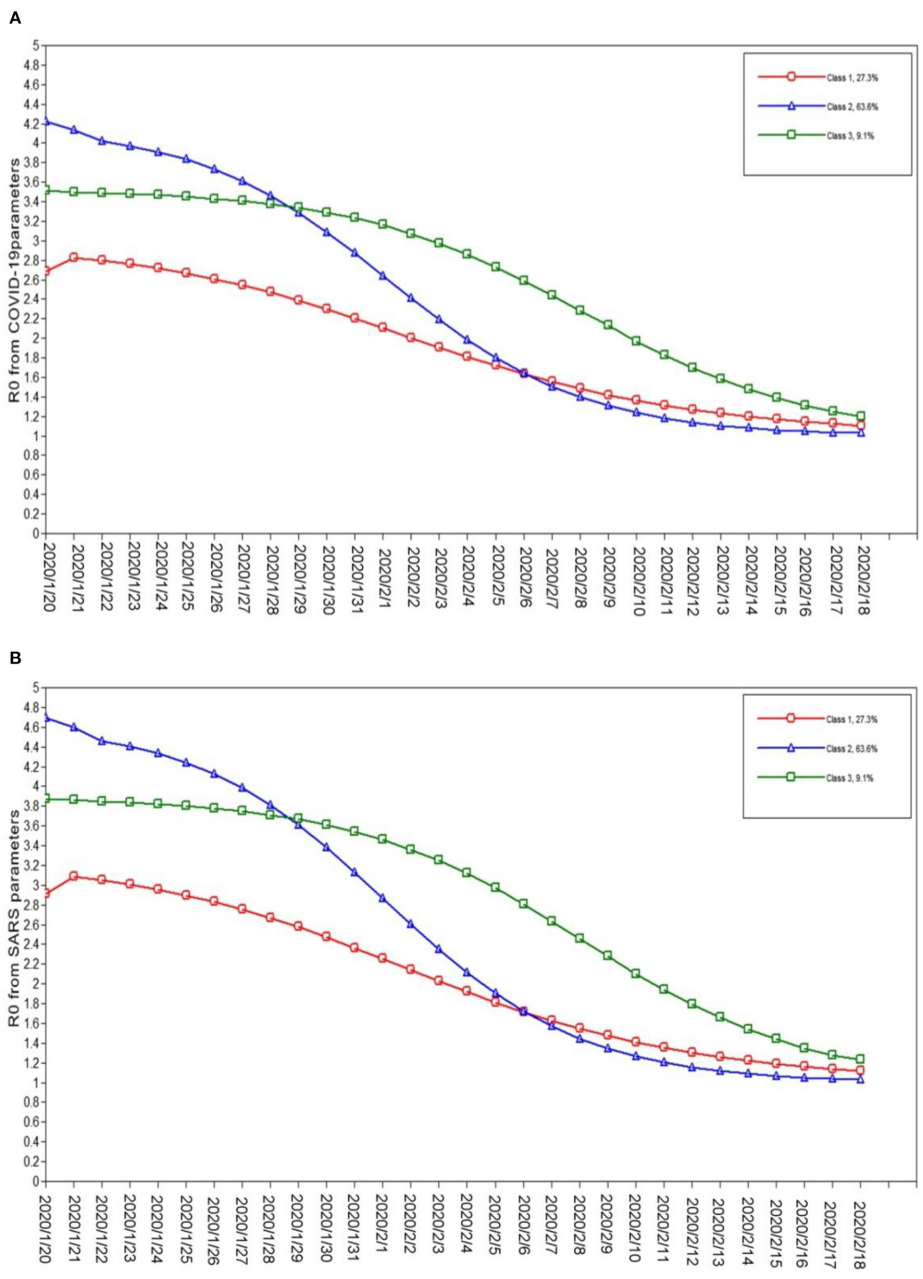
The latent class of *R*_0_ from COVID-19 and SARS parameters. **(A)** *R*_0_ from COVID-19 parameters everyday; **(B)** *R*_0_ from SARS parameters everyday.

We constructed five latent class models in the LPA. The three-class model was finally selected according to the model fitting index, latent class probability, and the interpretability of the model. Table 2 presents the fitting index and latent class probability of the model. There are three latent classes of the *R*_0_ calculated from the SARS parameters and COVID-19 parameters. Class 1 includes Shandong Province, Sichuan Province, and Chongqing Municipality. Class 2 includes Anhui Province, Hunan Province, Jiangxi Province, Henan Province, Zhejiang Province, Guangdong Province, and Jiangsu Province. Class 3 includes Hubei Province. Figure 4 shows the *R*_0_ trend in different latent classes. The initial value of class 1 *R*_0_ was relatively low and decreased slowly. The initial value of class 2 *R*_0_ was relatively high and decreased rapidly. The initial value of class 3 *R*_0_ was in the range between classes 1 and 2, but high *R*_0_ levels lasted longer and declined slowly.

**Table 2:** The fitting index and latent class probability of the model.

**R_**0**_**	**Model**	**AIC**	**BIC**	**aBIC**	**Entropy**	**Class probability (%)**
R_0_ from SARS parameters	1	317.51	341.38	160.72	-	1
	2	72.44	108.65	−165.35	1.00	18.2/81.8
	3	−92.09	−43.55	−410.89	1.00	27.3/63.6/9.1
	4	−30.09	30.79	−429.90	1.00	27.3/31.8/31.8/9.1
	5	−83.25	−10.04	−564.06	1.00	18.2/27.3/36.4/9.1/9.1
R_0_ from COVID-19 parameters	1	229.47	253.35	72.69	-	1
	2	−16.12	20.09	−253.91	1.00	18.2/81.8
	3	−181.98	−133.43	−500.78	1.00	27.3/63.6/9.1
	4	−119.98	−59.10	−519.78	1.00	27.3/63.6/4.5/4.5
	5	−149.15	−75.9	−629.96	1.00	0/27.3/36.4/27.3/9.1

## Discussion

The present study suggests that the *R*_0_ of COVID-19 calculated from the SARS parameters is greater than the *R*_0_ calculated from the COVID-19 parameters (the medians of *R*_0_ are 1.84–3.18 and 1.74–2.91, respectively). Our findings may indicate that the epidemiological characteristics of COVID-19 and SARS are different. Coronavirus has caused three large-scale epidemics within the human population in the past 20 years, including SARS in 2002, Middle East respiratory syndrome (MERS) in 2012, and COVID-19 in 2019 (28, 29). From the number of infections, deaths, and endemic areas, COVID-19 threatens human health more than SARS and MERS (30–32). The *R*_0_ of COVID-19 in our study is lower than that of SARS (3.1–4.2) and MERS (2.0–6.7) (17, 33, 34). These findings are consistent with previous studies, which also demonstrate that the infection capacity of COVID-19 is lower than that of SARS and MERS (23). Additionally, the *R*_0_ calculated from the COVID-19 parameters in this work is similar to the *R*_0_ (2. 38–2.72 and 2.47–2.86) estimated in other studies in China (20, 35). In addition, it is also similar to the *R*_0_ (2.4–2.8) of COVID-19 in Japan (36). Nevertheless, our calculated *R*_0_ is lower than the estimated *R*_0_ using the epidemic values observed in the early epidemic period of COVID-19 within Wuhan (*R*_0_ = 1.4–3.9), Hubei Province (*R*_0_ = 2.80–4.48), and China as a whole (2.8–3.3) (23, 37).

Overall, the *R*_0_ of COVID-19 in each province is gradually decreasing. It is believed that the combined prevention and control measures adopted for COVID-19 by the Chinese government have brought about these obvious effects. We believe these combined prevention and control measures for COVID-19, which are put in place by the Chinese Government, can provide a reference for other countries in their fight against the deadly virus. These measures include activating first-level public health emergency response, wearing masks, conducting epidemiological investigations, screening of key populations, temporary traffic control, closing public places (e.g., cinemas, internet cafe, etc.), monitoring body temperature, symptom screening, medical observation, the “four early” measures (early detection, early reporting, early isolation, and early treatment), the “four concentrated” treatment measures (concentrated cases, concentrated experts, concentrated resources, and concentrated treatment), quarantine oneself, lockdown a city, disinfection in public areas, etc. There is substantial evidence that implementing these combined measures could significantly reduce the number of cases found within a country (38, 39). A study conducted in Singapore showed that these combined interventions will reduce the estimated number of infections in comparison with the baseline scenario by 99.3%, 93.0%, and 78.2% when *R*_0_ was 1.5, 2.0, and 2.5, respectively (39). Some researchers suggest that the isolation of cases, contact tracing, and social distancing can control outbreaks of infectious diseases (38). Specifically, contact tracing is believed to be a key factor in reducing the spread of the epidemic. A new study from the UK suggests that to control the majority of COVID-19 outbreaks, for an *R*_0_ of 3.5 more than 90% of the contacts had to be traced, and for an *R*_0_ of 2.5 more than 70% of the contacts had to be traced (40).

The results of the LPA reported that there are three latent classes of *R*_0_, and the trends of *R*_0_ in each latent class have their own unique characteristics. The observed findings indicate that although the Chinese government has adopted the overall prevention and control measures of “a board of chess in China, suit one's measures to local conditions,” the effects are not entirely consistent. Specifically, the initial value of *R*_0_ in class 1 was relatively low and had a slow decline. The possible explanations are that these areas have taken active measures after the COVID-19 epidemic in Wuhan, and the spread of COVID-19 was well controlled from the beginning. The initial *R*_0_ value in class 2 was relatively high and was even higher than that of Hubei Province. However, *R*_0_ declined rapidly, and the decline rate was the fastest among the three latent classes. The possible reason is that the measures adopted at the beginning of the epidemic of COVID-19 were not effective. Then, the epidemic was effectively controlled after quickly adjusting the measures in these areas, thus the infection capacity of COVID-19 decreased rapidly. The initial *R*_0_ value in class 3 (Hubei Province) was observed to be between that of types 1 and 2, but this higher *R*_0_ level lasted longer and decreased slowly. Wuhan city, Hubei Province, is the first area in China where COVID-19 was found to be endemic. Due to the lack of understanding of the emerging infectious disease pathogens, transmission routes, susceptible populations, disease characteristics, epidemic characteristics, and other unknown reasons, the *R*_0_ of COVID-19 lasted longer at higher levels in Hubei Province. In other words, before January 31, 2020, Hubei Province COVID-19 was strongly infective. Therefore, our findings agree that the adoption of active prevention and control measures at the beginning of the epidemic can effectively control the spread of COVID-19.

What is more, the *R*_0_ of COVID-19 in Hubei Province and Shandong Province were approximately 1.2 by February 18, 2020, presenting that there is still a certain risk of transmission within these provinces. The *R*_0_ (1.01–1.06) in other provinces were approximately equal to 1 or greater than 1. It is widely recognized that *R*_0_ can reflect the endemic trend of infectious diseases. When *R*_0_ > 1, the greater the *R*_0_, the greater the infectious ability of these diseases, and the greater the number of cases. When *R*_0_ < 1, it means that the epidemic of infectious diseases will gradually come to an end. The optimal target of effective interventions is to control the *R*_0_ at a value < 1 (41). At present, because the population of China has gradually resumed work and school, population movement and crowd accumulation make it difficult to properly implement prevention and control measures, which ultimately increase the risks of COVID-19 transmissions within the country and also the difficulty of prevention and control. Furthermore, COVID-19 is an epidemic that has spread to many countries around the world, with international import cases giving rise to the possibility of a secondary epidemic (42, 43). Consequently, China also needs to continue to strengthen its prevention and control for COVID-19.

In this study, the *R*_0_ of COVID-19 was simulated by searching the confirmed cases and objective case data from the provinces closely related to Hubei Province (such as provinces with major transportation hubs and provinces with migrant workers moving within them) and to combine the methods mentioned in previous studies with the latent category method. The results of the three categories also show that the population distribution and economic conditions of different cities (20) also determine the infection rate and transmission rate of COVID-19. Despite the identification of human-to-human transmission and the reporting of exponential increases in the number of cases, forecasting is critical for national and international public health planning and control. At present, there is no good treatment available, however, timely understanding of the prevalence of COVID-19 with public awareness on preventative measures will help. Our study can provide the government with a more comprehensive chart of the epidemic trend and corresponding measures, and also provide side information for the storage of medical resources and clinical diagnosis and treatment. In China, COVID-19 in the regions other than Hubei Province showed a substantial control of the virus transmission rates. However, the possibility of imported infection and transmission was higher than before, in addition, because, at the time of this work, there was neither a vaccine nor a specific drug treatment for COVID-19, a range of public health (non-pharmaceutical) interventions has been used to control the epidemic (44).

The present study has several strengths and limitations. The main strength of this work was to calculate the *R*_0_ based on the epidemic data of major endemic areas of China as of February 18, 2020. However, previous research on *R*_0_ was mostly predicted and estimated based on the limited epidemiological data of COVID-19 in Wuhan in the early days. Another strength was an analysis of the latent class of *R*_0_ and explored the regional differences in prevention and control effects. The findings presented in this study have implications for the preliminary evaluation of the effectiveness of prevention and control measures for COVID-19 adopted in China. In addition, the research results can provide knowledge for other countries on how to respond to COVID-19, and also provide a support for previous prediction studies. There are several limitations to our study. Firstly, the calculation of *R*_0_ was affected by multiple parameters and various factors (41). And yet, the factors considered are relatively single in this study. Additional research is necessary to confirm the accuracy of *R*_0_, considering the large uncertainties around the estimates of *R*_0_ and the duration of infectiousness (45, 46). Secondly, there are many methods for calculating *R*_0_ in the world (18), but this study did not use multiple methods to compare the results. In the susceptible-exposed-infected-removed model, the *R*_0_ of COVID-19 was a little higher than that of SARS (23). Wang et al. (37) used the exponential growth method to estimate *R*_0_, the results showed that the *R*_0_ of COVID-19 was 2.95 (95%CI: 2.86–3.03) after taking control measures. One study used a susceptible-exposed-infectious-recovered metapopulation model to simulate the *R*_0_ for COVID-19, which was 2.68 (95%CI: 2.47–2.86) (20). In our study, we choose a new-structure gray Verhulst model that has a better structure and stronger modeling ability; overcoming the shortcomings of traditional Verhulst model, including parameter dislocation and unreasonable selection of initial values. We used another method to supplement the previous study, which laterally validated and supplemented the previous results. However, it should be noted that if other methods are used to calculate *R*_0_ in this study, the results may be different. Thirdly, although this study discusses the prevention and control measures for COVID-19 in the major endemic areas of China, it is difficult to quantitatively evaluate the effects of various measures.

## Conclusion

Overall, we found that the *R*_0_ of COVID-19 shows a downward trend in the major endemic areas of China, and there are regional differences (three latent classes). Actively adoption of combined prevention and control measures in the early stages of the epidemic can effectively control COVID-19.

## Data Availability Statement

The datasets presented in this study can be found in online repositories. The names of the repository/repositories and accession number(s) can be found in the article/Supplementary Material.

## Ethics Statement

Ethical approval was not required as this study is an analysis of public case data. The patients/participants provided their written informed consent to participate in this study.

## Author Contributions

FT designed the study. HX took primary responsibility for writing the manuscript, managed the literature searches and analyses, and undertook the statistical analysis. MY, LM, ML, YZ, WL, and HG undertook the acquisition of the data. YZ revised the manuscript. GL edited the English Version of this manuscript. All authors contributed to and approved the final manuscript.

## Funding

This work was supported by the Anhui Medical University Emergency Key Research Project for Novel Coronavirus Pneumonia (YJGG202001) and Emergency Research Project, Anhui Provincial Department of Science and Technology, Anhui Provincial Health Commission (202004a07020002).

## Conflict of Interest

The authors declare that the research was conducted in the absence of any commercial or financial relationships that could be construed as a potential conflict of interest.

## Publisher's Note

All claims expressed in this article are solely those of the authors and do not necessarily represent those of their affiliated organizations, or those of the publisher, the editors and the reviewers. Any product that may be evaluated in this article, or claim that may be made by its manufacturer, is not guaranteed or endorsed by the publisher.
